# Computational Methods for Assessing Chromatin Hierarchy

**DOI:** 10.1016/j.csbj.2018.02.003

**Published:** 2018-02-15

**Authors:** Pearl Chang, Moloya Gohain, Ming-Ren Yen, Pao-Yang Chen

**Affiliations:** Institute of Plant and Microbial Biology, Academia Sinica, Taipei, Taiwan

**Keywords:** 3D genome, Chromatin accessibility, Chromosome conformation capture, 3C-technologies, Hi-C, ATAC-seq

## Abstract

The hierarchical organization of chromatin is known to associate with diverse cellular functions; however, the precise mechanisms and the 3D structure remain to be determined. With recent advances in high-throughput next generation sequencing (NGS) techniques, genome-wide profiling of chromatin structures is made possible. Here, we provide a comprehensive overview of NGS-based methods for profiling “higher-order” and “primary-order” chromatin structures from both experimental and computational aspects. Experimental requirements and considerations specific for each method were highlighted. For computational analysis, we summarized a common analysis strategy for both levels of chromatin assessment, focusing on the characteristic computing steps and the tools. The recently developed single-cell level techniques based on Hi-C and ATAC-seq present great potential to reveal cell-to-cell variability in chromosome architecture. A brief discussion on these methods in terms of experimental and data analysis features is included. We also touch upon the biological relevance of chromatin organization and how the combination with other techniques uncovers the underlying mechanisms. We conclude with a summary and our prospects on necessary improvements of currently available methods in order to advance understanding of chromatin hierarchy. Our review brings together the analyses of both higher- and primary-order chromatin structures, and serves as a roadmap when choosing appropriate experimental and computational methods for assessing chromatin hierarchy.

## 1 Introduction

**Fig. 1:**
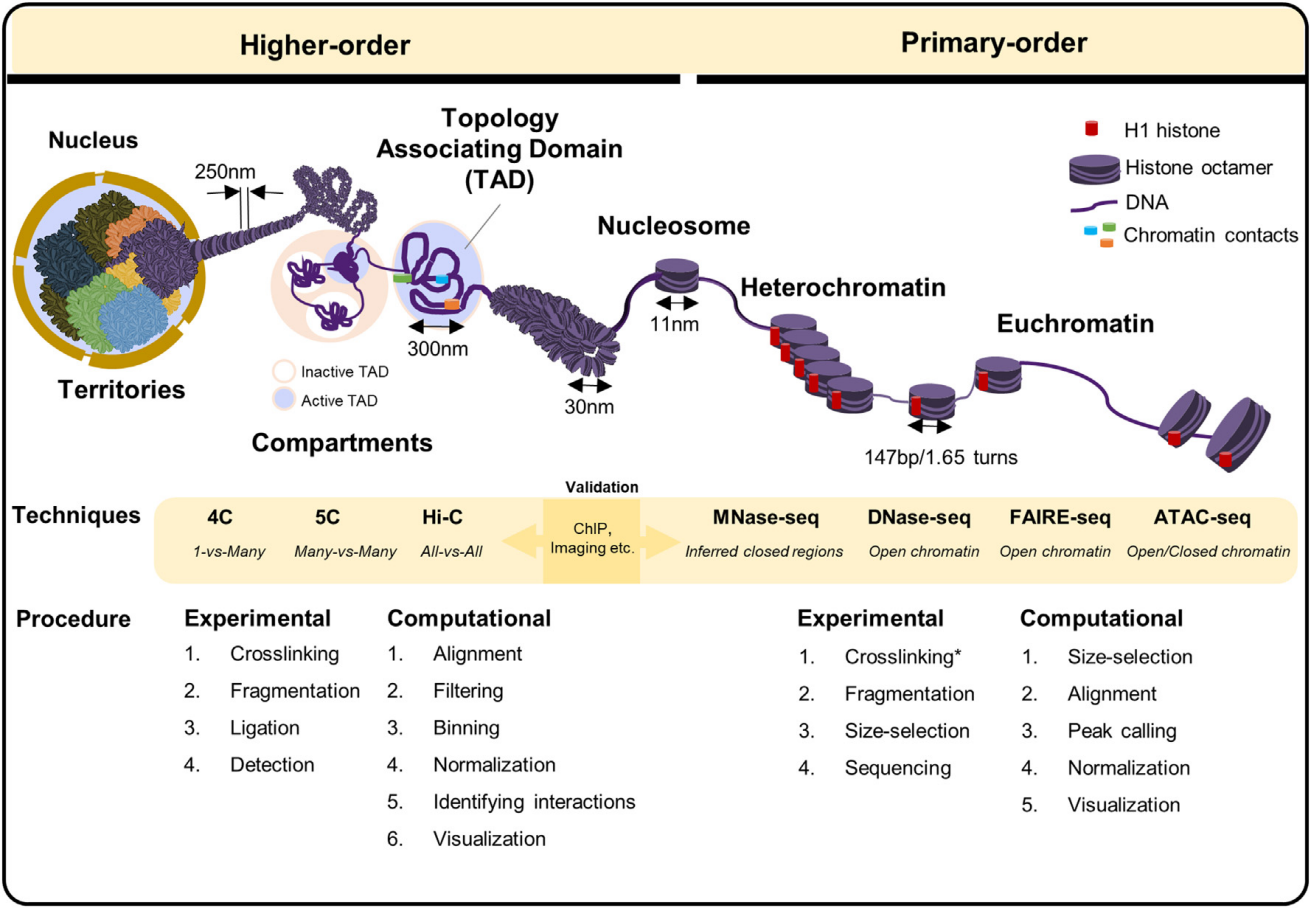
Genome organization in eukaryotes from higher to primary orders. Features of chromatin organization from higher- to primary-order. Techniques, experimental and computational procedures for assessment of chromatin hierarchy. The active circle represents TADs rich in genes and show early replication. The inactive circle represents TADs that harbor few genes and show late replication. *Among the chromatin accessibility profiling methods, only FAIRE-seq strictly requires crosslinking.

Chromatin is a compact and organized assembly of DNA and proteins [32] that is intricately folded into three dimensions, forming different levels of organization in the nucleus. The highest order of chromatin organization is visible during cell division as a chromosome. In a mammalian chromosome, DNA is condensed approximately 10,000 to 20,000-fold [58], and the structure of chromosomal DNA can be categorized as “higher-order” and “primary-order” according to the folding complexity (See Fig. 1 for an overview and assessment of the hierarchical organization of chromatin).

The higher-order genome structure is most clearly visible during the interphase and mitosis when chromatin fibers extensively fold into chromosomes. An interphase chromosome is formed by a tightly coiled 250 nm chromatid. Microscopic imaging has demonstrated that each chromosome may be confined to genomic compartments [59]. Within these compartments, intra-chromosomal interactions are most frequent within regions known as megabase-sized topologically associating domains (TADs). The active TADs are rich in genes, open chromatin marks, transcription factors and DNase I-hypersensitive sites (DHSs) and show early replication. In contrast, the inactive TADs harbor few genes and DHSs and show late replication [77,80,91].

On the other hand, the primary-order chromatin refers to the unpacked chromatin fiber where 11-nm coils of nucleosomes are exposed. The nucleosome is the fundamental unit of chromatin. Each nucleosome comprises 147 bp of DNA wound 1.65 times around core histones [54,74]. Chromatin can be categorized into two varieties: euchromatin and heterochromatin [31]. They differ in terms of the overall compaction of nucleosomes, numbers of genes and transcription levels. The loosely packed regions form the “euchromatin”, whereas the densely-packed regions form the “heterochromatin” and represent the less accessible part of the genome [5]. Typically, euchromatin is enriched in genes, and transcription in this region is active. Heterochromatin usually consists of repetitive sequences and forms structures such as centromeres. However, the condensed structure of some heterochromatin can become loose and transcription may take place when under certain developmental or environmental conditions [38,45].

Gene expression and biological functions intimately rely on the interactions between regions (higher-order structure) and the accessibility of chromatin (primary-order structure), which are mediated by protein complexes and epigenetic modifications [7,79,88]. The set of chromatin-associated proteins and epigenetic modifications at a given time in a genomic region constitutes the “chromatin state”. With the latest sequencing techniques followed by computational analysis, it is now possible to detect chromatin interactions and its accessibility in the context of functional significance.

This review aims to give a broad overview of NGS-based methods for “higher-order” and “primary-order” chromatin assessment from both experimental and computational aspects. We discuss the characteristics and requirements of each sequencing method together with the computing strategies and bioinformatics tools.

### 1.1 Assessment of Higher-order Chromatin Structure

#### 1.1.1 Experimental Techniques for the Assessment of Higher-order Chromatin

Microscopy-based imaging tools have been used to observe the higher-order structure of chromatin and its dynamics for over a century [42]. At a resolution of 50–100 nm, light microscopy reveals the shape and distribution of chromosomes in single cells but fails to provide comprehensive detail of the spatial interactions [46]. The development of electron microscopy (EM) and fluorescence *in situ* hybridization (FISH) have provided evidence of chromosomal territories and compartments, organization of TADs and non-random organization of genomic loci within the nuclear periphery [71,104].

Over the past decade, a variety of chromosome conformation capture (3C)-based methods have allowed the detection of higher-order structures of chromatin in unprecedented detail. The conventional 3C method determines the physical interactions of chromatin between two genomic regions (one vs. one) [30,84,102]. The experimental steps include formaldehyde crosslinking to fix *in vivo* contacts, chromatin fragmentation by restriction enzyme digestion and proximity ligation of the digested ends. The restriction enzyme selection depends on the size of target loci; for 3C, frequently cutting enzymes give rise to smaller fragments and hence are more suitable for identifying smaller loci. As a guideline, 4-bp cutters (i.e. frequent cutters) are used when studying small loci sized below 10–20 kb, whereas 6-bp cutters are for loci larger than 20 kb. Ligation junctions are detected in conventional 3C libraries via PCR followed by gel electrophoresis. In combination with next-generation sequencing, the physical interactions of chromatin can be detected with a higher resolution and greater sensitivity [33,56].

More recent 3C-based technologies, such as 4C, 5C, and Hi-C, incorporate next generation sequencing and thereby are capable of providing quantitative measurements for intra (*cis*)- and inter (*trans*)-chromosomal interactions. Circular chromosome conformation capture (4C) uses restriction digestion, followed by inverse PCR, to identify multiple loci interacting with one particular genomic site, referred to as the “bait” or “viewpoint” (one vs. all) [89,93]. The size of a viewpoint is dependent on the primary restriction enzyme used. The optimal size of a viewpoint is approximately 1 kb; viewpoints larger than 1 kb tend to have difficulties to form ligated products, whereas viewpoints that are too short suffer from a lower probability to detect interactions [98]. Furthermore, the reliability of identified close-range (*cis*) or long-range (far-*cis* or *trans*) contact sites depends on experimental setups. Analyses resulted from 4-bp cutter enzymes have been shown to have low reproducibility of 4C signals between replicates, particularly in far-*cis* and *trans* interactions; however, 4-bp cutters are effective in identifying *cis* interacting loci in the vicinity (<10 kb) of the viewpoint [35,99]. In comparison to 4-bp cutters, 6-bp cutters have proven effective in characterizing reliable interactions in distance ranging from 10 kb to 10 Mb [27,73,75]. For extremely long distance interaction (>10 Mb), the signal-to-noise ratios can be improved by *in situ* ligation that occurs inside the nuclei instead of “in solution,” thereby decreasing the probability of false inter-chromosomal fusions [98].

Chromosome conformation capture carbon copy (5C) is employed to study all contacts within a particular region (many vs. many), based on highly multiplexed ligation-mediated amplification (LMA) [87]. This technique uses primer pairs that anneal on either side of all ligation junctions in the region of interest in a 3C-based library. These fragments are amplified in a single amplification reaction, which can be analyzed using microarrays or high-throughput sequencing.

Hi-C generates contact maps among all parts of the genome (all vs. all) [78]. A biotin-labeled nucleotide is filled in after fragmentation, followed by blunt-end ligation. An enrichment step via streptavidin bead pull-down concentrates ligation junctions, which are subsequently analyzed using high-throughput sequencing. The Hi-C technique eliminates the need to design specific oligo primers and also increases the resolution to ~1 Mb with 10 million pair-end reads [60]. Its resolution though is difficult to be further improved since a 10-fold increase in resolution requires a 100-fold increase in sequence depth [27]. Therefore, Hi-C can only resolve on the Mb level for most multicellular organisms and correlation with specific genes or epigenetic marks still remains implausible. Nevertheless, Hi-C still is a powerful tool for revealing chromosome territories and genome compartmentalization. Table 1 highlights the workflow, data analysis, experimental requirements, resolution, advantages and drawbacks common to 3C-based technologies.

**Table 1:** Techniques for assessment of higher-order and primary chromatin structure.

Techniques	Target	Method	Requirements	Resolution	Pros and cons	Reference
*Higher-order*						
Non-NGS-based method						
3C	1 -vs-1	-Cross-linking -Fragmentation -Intra-molecular ligation -Reverse crosslink -Purification -qPCR detection	-2 × 10^7^–2.5 × 10^7^ cells -Primer: long, high Tm, unidirectional	~1–10 kb	**Pros** High dynamic range, quantitative, easy data analysis **Cons** Cannot detect novel contacts Low throughput	[108]

NGS-based method						
4C	1-vs-All	-Cross-linking -Fragmentation -Immunoprecipitation -Re-ligation -Enrichment -Amplification -Microarray/NGS	−4 bp-cutter -Inverse PCR-sequencing -Min. Reads: 1–2 million (human)	~10 Mb	**Pros** Detects novel contacts, high resolution, sensitivity for long-range contacts, high-throughput, reproducible **Cons** Limited to unique viewpoint	[90,109]
5C	Many-vs-Many	-Cross-linking -Fragmentation -Immunoprecipitation -Re-ligation -PCR/sequencing	Multiplexed LMA sequencing -Min. Reads: 25 million (human)	~4 kb	**Pros** High dynamic range, complete contact map of a locus, overcomes junctional problems **Cons** Probe bias, limited to the selected region	[90,110]
Hi-C	All-vs-All	-Cross-linking -Fragmentation -Biotin labeling -Re-ligation -Streptavidin binding -Shearing -Sequencing	−300-500 bp fragment - 8.4 to 100 million reads (human) -2 × 10^7^–2.5 × 10^7^ cells	~1 Mb	**Pros** Detects all intra- and inter-chromosomal interactions **Cons** High cost	[60,90]

*Primary-order*						
NGS-based method						
MNase-seq	Nucleosomes; Inferred closed regions	-Cross-linking (optional) -MNase digestion -Size selection -Sequencing	-Size selection: 25–200 bp -Paired-end or Single-end -Min. Reads: 150 to 200 million (human) - 10^6^–10^7^ cells	~ 1–10 bp	**Pros** Detects TF footprints, method of choice for genome-wide nucleosome core positioning **Cons** Accessible regions are indirectly inferred, large numbers of reads for sufficient depth, MNase sequence bias	[111]
DNase-seq	Open chromatin	-Cross-linking (optional) -DNase I digestion -Size selection -Sequencing	-Size selection: 50–100 bp -Paired-end or Single-end -Min. Reads: 20 to 50 million (human) −10^6^–10^7^ cells	~1 bp	**Pros** Detects TF footprints, greater sensitivity at promoters than FAIRE-seq **Cons** Time-consuming, DNase I sequence bias	[112]
FAIRE-seq	Open chromatin	-Cross-linking -Sonication -Phenol-chloroform extraction -Reverse cross-linking -Sequencing	-Paired-end or single-end -Min. Reads: 20 to 50 million (human) - 10^5^ - 10^7^ cells	~200 bp	**Pros** Simple experimental procedure **Cons** Variable crosslink efficiency, lower resolution, high noise-to-signal ratio, complicated computation and interpretation	[113]
ATAC-seq	Open/closed chromatin	-Fresh nuclei isolation in most cases -Tn5 transposition -Sequencing	-Paired-end -Min. Reads: 100 to 160 million (human) -5 × 10^2^ to 5 × 10^4^ cells	~1 bp	**Pros** Simple, fast sample preparation, lower input, detects TF footprints, detects nucleosome occupancy **Cons** Fresh tissue isolation, mitochondrial DNA contamination, sequence bias of Tn5 transposase, immature data analysis tools	[16]

#### 1.1.2 Computational Approaches for Assessing Higher-order Chromatin

**Fig. 2:**
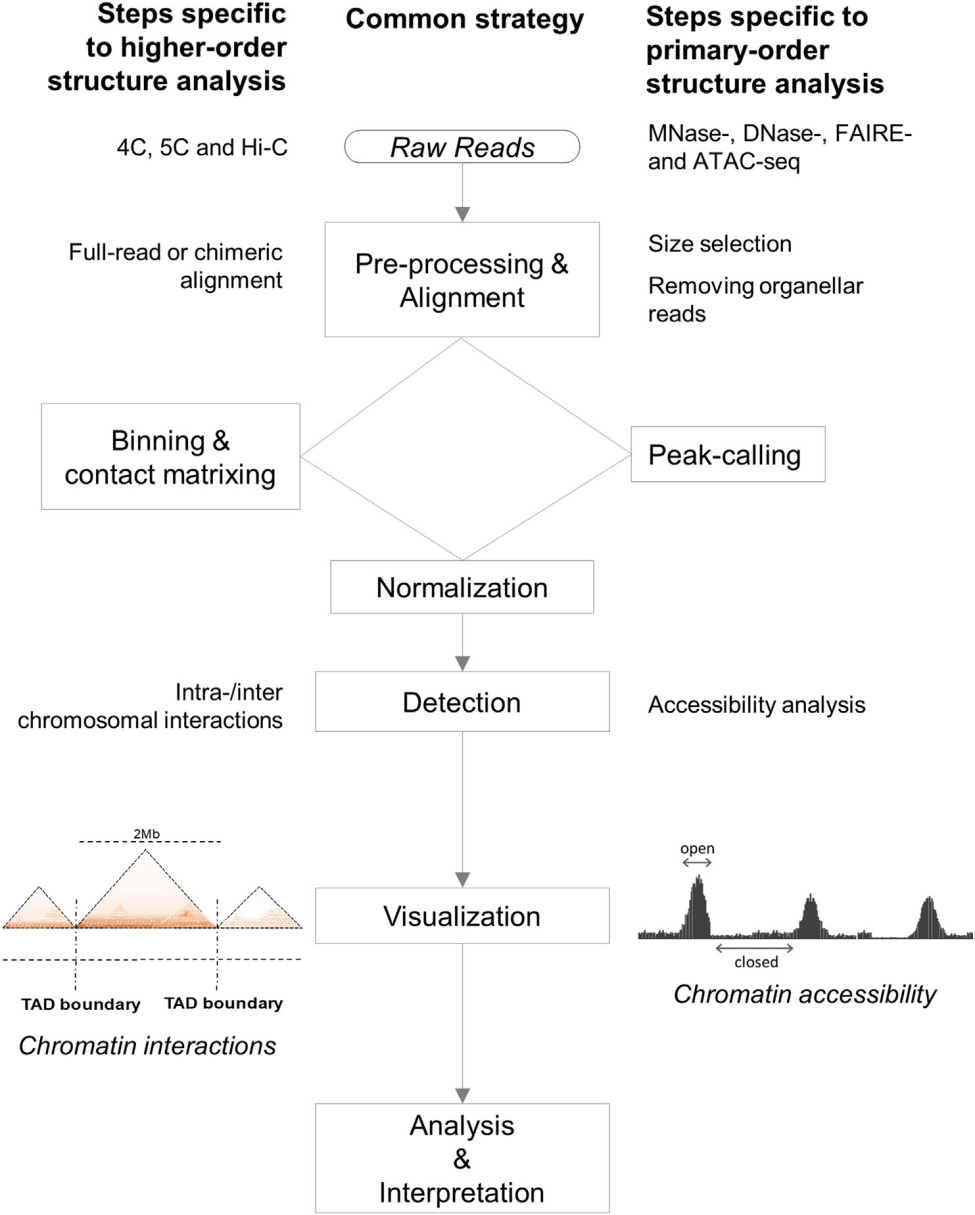
Common computational analysis strategy and specific steps for assessing higher-order and primary-order chromatin structures. The common computational steps are outlined in the center. Steps specific to the higher-order or primary-order analysis are indicated on each side. The raw reads from 3C-based techniques follow the pipeline to the left to reveal higher-order chromatin interactions. The raw reads for chromatin accessibility analysis follow the pipeline to the right.

The advancement of 3C-based technologies and rapid accumulation of data challenge the computational analysis and interpretation. Here, we describe the key features of 3C-based data analysis, with key steps outlined in Fig. 2. For the detection of chromatin interactions using high-throughput sequencing, the general steps in the analytical pipeline start with the preprocessing of paired-end raw reads. After quality filtering based on Phred scores and user-defined filters, the remaining reads are mapped to the genome of interest via alignment strategies. Appropriate bin size is then selected according to the distance between interacting sites, followed by normalization that reduces bias and enables comparisons between different samples. Identification of intra- and inter-chromosomal interactions then is determined with visualization. Specific tools for each of these steps are listed in Table 2.

**Table 2:** Computational tools for the assessment of chromatin hierarchy.

Tools		Function	References
***Common to both higher- and primary-order assessment***			
**Aligners** Bowtie2 BWA SOAP RMAP Cloudburst SHRiMP		-Ultrafast, sensitive, accurate and memory-efficient gapped read aligner. -Maps low-divergent sequences against a large reference genome. -Efficient gapped and ungapped alignment of short oligonucleotides to reference. -Maps reads from short-read sequencing technology. -Parallel read-mapping algorithm optimized for mapping NGS data. -Fully gapped local alignment of short reads to targets.	[114] [58] [59] [91] [80] [77]

***Higher-order***			
**4C** FourCSeq		-Uses R to detect specific interactions between DNA elements and identify differential interactions between conditions.	[115]
**5C** HiFive		-A Python package for normalization and analysis of chromatin structural data produced using either the 5C of HiC assay.	[79]
**Hi-C** Fit-Hi-C GOTHiC HOMER HIPPIE HiCCUPS HiCPipe Juicer		-Assigns statistical confidence to mid-range *cis*-chromosomal contacts. -Models contact-frequency uncertainty as binomial distribution. -Designed for high-resolution Hi-C data. -Identifies chromatin interactions in a genome. -Detect sub-TAD chromatin interactions (*cis*). -Provides scripts and programs that correct Hi-C contact maps. -Aligns, filters and normalizes, identifies and compares TADs, loops and compartments and display using Juicebox.	[7] [116] [41,42] [46] [71] [104] [29,30]
HiGlass		-Enables multiscale navigation of TAD interactions along with 1D genomic tracks	[52]
**TAD calling** TADbit TADtree Armatus		-TADbit includes quality control module, and aligns reads to the reference. -Identifies hierarchical topological domains. -Uses dynamic programming to call TADs in different resolutions.	[84] [102] [33]

***Primary-order***			
**Primary assessment** ArchTEX DANPOS-profile CEAS Artemis EagleView Integrative Genomics Viewer		-Java-based tool for identification of optimal extension of sequence tags. -Dynamic nucleosome analysis at single-nucleotide resolution. -Provides statistics on fragment enrichment in important genomic regions. -Java-based free genome browser, annotation and visualization tool. -Viewer for next-generation genome assembles with data integration capability. -Lightweight visualization tool for intuitive real-time exploration of diverse data.	[56] [19] [87] [78] [117] [118]
**Peak-calling** MNase-seq	GeneTrack iNPS DANPOS	-Employs Gaussian smoothing for nucleosome calling. -Detects nucleosomes from the first derivative of the Gaussian smoothed profile. -Allows comparison of datasets and identification of dynamic nucleosomes.	[4] [20] [19]
DNase-seq	MACS2 Hotspot F-seq ZINBA	-Models length of DNA fragments for spatial resolution of predicted binding sites. -Identifies regions of local enrichment of short-read sequence tags. -Identifies chromatin accessible regions and tentative TF footprints. -Generates peak calls that are consistent with known biological patterns.	[106] [49] [14,15] [72]
FAIRE-seq	MACS2 ZINBA	-Models length of DNA fragments for spatial resolution of predicted binding site. -Generates peak calls that are consistent with known biological patterns.	[106] [72]
ATAC-seq	MACS2 Hotspot HOMER F-seq ZINBA	-Models length of DNA fragments for spatial resolution of predicted binding site. -Identifies regions of local enrichment of short-read sequence tags. -Motif discovery and transcript identification analysis. -Identifies chromatin accessible regions and tentative TF footprints. -Generates peak calls that are consistent with known biological patterns.	[14,15] [106] [49] [41,42] [72]
**Accessibility analysis** CENTIPEDE V-Plots DNase2TF		-Infers regions of the genome bound by transcription factors. -Plots to reveal chromatin features of transcription factor binding sites. -Footprinting algorithm with accurate detection and less computing time.	[67,68] [43] [94]

##### 1.1.2.1 Pre-processing and Alignment

Raw reads are pre-processed by filtering out PCR duplicates and potential artifact reads reduces false-positive signals. Sequencing adaptors can also be removed prior to alignment. There are two types of alignment strategies, the full-read approach and chimeric alignment. The full read alignment method employs standard alignment software such as Bowtie2 [57] or the Burrows-Wheeler Aligner (BWA) [58], with which read pairs are independently aligned to a reference genome using an end-to-end approach. The unmapped reads from full-read alignment are mainly composed of chimeric fragments spanning the ligation junction. In order to rescue those unmapped reads, the chimeric alignment can be performed with read splitting [83] or iterative mapping [47]. Since chimeric alignment method is capable of identifying the different alignment positions of the sequences at two sides of the junction, chimeric alignment usually maps more reads than the full-read approach which cannot align reads spanning across the junction sequence. The difference in the proportion of mapped reads between these two alignment approaches becomes more apparent when the read length increases. In order to reach proper coverage and depth, sufficient mapable sequencing reads must be obtained. In the case of human genome, Sims et al. [90] summarized the minimal numbers of reads for 4C (1–2 million), 5C (25 million) and Hi-C (8.4 to 100 million) (Table 1). Among those, Hi-C needs the most number of reads in order to identify interactions between all possible sites in the whole genome.

##### 1.1.2.2 Binning and Generating Contact Matrices

In 3C analysis, the signal-to-noise ratio decreases with increased distance between two target loci. To overcome this limitation, binning is employed in more advanced 3C-based techniques. A bin is a fixed, non-overlapping genomic span into which reads are grouped to increase the signal of the interaction frequency. Smaller bins usually are used for more frequent intra-chromosomal interactions, and larger bins are for less frequent inter-chromosomal interactions [12]. As a general rule, selected bin size should be inversely proportional to the expected number of interactions in a region. For long range chromatin interactions, binning may reduce the complexity resulted from local interactions. Filtering out bins with fewer interactions can also improve the signal strength. Such bins normally occur in regions with low mappability or high repeat content. The interactions between bins are simply summed up to aggregate the signal thus, reducing biases to infer a meaningful interaction profile from weak raw signals. The above are examples of how choosing a proper bin size is critical for data analysis. For the data to be reported without binning, there should be a sufficient signal strength and reproducibility at the level of individual restriction fragments. Hi-C read counts are typically summarized at the level of genomic bins with a fixed width. The range of the bin size varies from 5 kb to 1 Mb. With a determined bin size, the interaction frequency is stored as a contact matrix. The contact matrix is symmetric and two-dimensional, with each entry representing contact frequency between two genomic bins.

##### 1.1.2.3 Normalization

Several biases arise as a result of the experimental steps. The goal of normalization is to reduce such biases. Normalization also enables the direct comparison of data from different replicates and conditions on a common scale. There are two general approaches for bias correction: explicit and implicit normalization. Explicit models take into account of known bias factors. In Hi-C experiments as an example, several systematic biases influence the Hi-C read counts, including the distance between restriction enzyme cut sites, the GC content of trimmed ligation junction and uniqueness of sequence reads [104]. In order to remove these systematic biases, several approaches, such as integrated probabilistic background model and Poisson regression model can be used for data normalization [44,104]. Since it is improbable to include all bias factors, alternatively is the use an implicit approach, also known as iterative correction [47]. This procedure corrects the matrix by equalizing the sum of every row and column in the matrix. The procedure is based on the assumption that all loci should have equal visibility since we are detecting the entire genome in an unbiased manner. This implicit, iterative correction algorithm, is relatively faster and therefore preferred.

##### 1.1.2.4 Identification of Intra- and Inter-chromosomal Interactions

The most popular and intuitive algorithms for identifying intra-chromosomal interactions, known as TADs, include the directionality index (DI) [28] and the insulation index (ID) [119]). DI is a statistic for quantifying the degree of upstream or downstream interaction bias in the genome and varies considerably around TADs [28]. It is calculated for individual bins by collecting the reads that fall into the bin and observing whether the paired reads are mapped upstream or downstream of the bin. A positive DI indicates downstream bias of the read pairs. Based on DI, TAD boundaries are demarcated by strong directionally biased loci. In contrast, ID uses a sliding window approach to sum up contacts within a given region surrounding each locus [49]. TADs are demarcated by boundaries consisting of insulators that impede DNA contacts across nearby domains. ID sums up contacts within a given region surrounding each locus - as TADs are regions of increased contacts, they can easily be identified via contact count cutoffs. Tools for identifying TADs are referred to as TAD callers.

Most TAD calling tools have the options for both DI and ID for TAD calling. For example, TADtool (as a Python package) enables the direct export of TADs called using a set of parameters for both directionality and insulation indices [14]. Other TAD callers, such as TADbit [72], Armatus [33], and TADtree [72], exhibit balanced performance for most parameters for experimental and simulated data. Interaction callers, such as HOMER [14] and HiCCUPS, [30,71] yield the highest proportion of biologically significant chromatin interactions. HiCCUPS maps Hi-C data to a specified reference genome and removes artifacts but does not perform genome binning and normalization and requires other tools, such as HiCPipe [104], for downstream processing.

Most research in this area has focused on the interactions within individual chromosomes, and those between different chromosomes have received less attention. A major challenge is the identification of reliable and reproducible inter-chromosomal contacts; for example, false inter-chromosomal fragments could result from random ligations and in which case the contact profile shows an enrichment of inter-chromosomal interactions and a depletion of intra-chromosomal ones [44]. As the signal-to-noise ratio for long range contacts is lower [98], the inter-chromosomal contact analyses must be handled carefully and the results interpreted with caution. One strategy to search for inter-chromosomal interactions is the use of binary contact matrices. For instance, on a Hi-C dataset the interactions between numerous sites were first simplified into binary matrices with a cutoff for the interaction probabilities [50,55]. These binary contact maps were then mathematically transformed into inter-chromosomal segment interaction networks. Using this method, Kaufmann et al. [50] found a strong non-random clustering in both human and mouse genomes. Both genomes exhibit similar structural characteristics such as increased flexibility of specific Y chromosome regions and co-localization of centromere-proximal region [50]. This characterization of common structural properties between species points to new regulatory mechanisms based on the spatial distances between different chromosomes.

Hi-C dataset analysis requires powerful computers with high computing capacity as numerous interactions between all loci are examined. Although standard computers equipped with high-performance specs are usually sufficient for standard Hi-C analysis, some software packages require specialized hardware. For instance, HiCCUPS requires a general-purpose graphics processing unit (GPU) due to the large number of pixels (trillions) in a kilobase-resolution Hi-C map [29]. In some cases, specialized hardware is not required but could greatly accelerate the process. In a study comparing the performance of four different cluster systems that process 1.5 billion paired-end Hi-C reads using Juicer [71,104], the total required times varied from ~12,000 to ~600 h. This 20-fold increase in computing efficiency was achieved largely by incorporating general-purpose graphics processing units and field-programmable gate arrays (FPGAs) in the setup [30].

##### 1.1.2.5 Visualization

Chromatin interactions can be visualized as a heatmap in which the x- and y-axes represent loci in genomic order, and each pixel is the number of observed interactions between them. Plotting contact probabilities versus genomic distance typically reveals an inverse relationship between these two parameters. Hi-C data can be visualized using Juicebox [29], my5C [72] and 3D genome browsers [67] and HiGlass [52]. Epigenome Browser combines web technology with intuitive graphical design to visualize long-range interaction data [43,107]. Beside chromatin interaction data, epigenome browsers allow visualization of other omics data such as RNA-Seq, WGBS or ChIP-seq for a genomic region, providing a complete view of regulatory landscape and 3D genome structure for a given gene.

### 1.2 Assessment of Primary-order Chromatin

The spatial organization of the genome, and thus, cellular functions are also regulated at the primary scale. Chromatin compaction is determined by nucleosome density. Genomic regions with dense nucleosomes are more tightly packed (i.e., “closed”), whereas nucleosome-depleted regions are more accessible (i.e., “open”) for interactions with regulators and are therefore regarded as the primary locations of regulatory elements. Currently, NGS enables genome-wide investigations of chromatin accessibility [63,86,97]. In this section, we provide an overview of the common methods for profiling genome-wide chromatin accessibility and the data analyses involved. A comparison of these methods, in terms of experimental requirements and specificities, is shown in Table 1. An overall computational analysis strategy is outlined in Fig. 2, and specific bioinformatics tools are listed in Table 2.

#### 1.2.1 Experimental Techniques for Assessing Primary-order Chromatin

Currently, the most widely used methods for assessing primary-order chromatin state include MNase-seq, DNase-seq, FAIRE-seq and ATAC-seq (Fig. 1). For MNase-seq, an endo-exonuclease (MNase) that cleaves linker DNA between nucleosomes, with its endonuclease activity digesting linker DNA unprotected by the nucleosome core, resulting in nucleosome-bound DNA sequences. DNA regions with a high density of MNase-seq reads represent nucleosome-dense, tightly packed, closed chromatin [9,40,81]. Currently, MNase-seq is the method of choice for probing genome-wide nucleosome positioning [23]. It is noteworthy to point out that although euchromatic and heterochromatic regions are both accessible to MNase digestion, heterochromatin tends to give rise to longer, multiple nucleosome-sized fragments that can be excluded by size selection prior to MNase-seq analysis [105]. Hence, in standard MNase-seq data analysis, the reads included are usually predominantly from euchromatic regions, and higher MNase-seq read abundance represents the relatively small and closed regions in euchromatin. The proportion of euchromatin reads to heterochromatin reads varies with factors such as the chromatin state, enzyme digestion condition, and sequence read size selection limit.

In contrast, DNase-seq was developed to identify open chromatin regions based on the notion that accessible regions of the genome show hypersensitivity to DNase I endonuclease [39]. Upon endonuclease digestion, open regions that are unprotected by nucleosomes are cleaved into sub-nucleosomal fragments (<150 bp). These two enzyme-based methods can also be employed to identify transcriptional factor-bound DNA regions at a nucleotide resolution using libraries with subnucleosome-sized fragments down to 25 bp facilitates the identification of both nucleosomes and transcription factor (TF) binding sites [43,94,100].

FAIRE (formaldehyde-assisted isolation of regulatory elements)-seq [36,92] is a method for identifying open regions in the genome. DNA is crosslinked to nucleosomes using formaldehyde, which is subsequently removed by phenol-chloroform extraction. The remaining nucleosome-free DNA is sequenced to profile accessible regions. This experimental procedure is relatively simple but generally yields a lower resolution and high noise-to-signal ratio.

ATAC (assay of transposase-accessible chromatin)-seq identifies open and closed chromatin. The Tn5 transposase cleaves DNA fragments from open chromatin regions. After cleavage, Tn5 inserts adaptor sequences into integrated sites, eliminating additional ligation steps prior to sequencing. In addition to a simplified sample processing procedure, ATAC-seq generally requires two to four orders of magnitude fewer tissues/cells (see Table 1). Most ATAC-seq experiments have been performed on native (not crosslinked) cells, yet it was recently reported that formaldehyde fixation does not affect the Tn5 tagmentation efficiency in intact nuclei [21]. An alternative to profile accessible regions for fixed cells is NicE-seq (nicking enzyme assisted sequencing). A nicking enzyme targets open chromatin and these open regions are labeled with biotin due to the incorporation of biotinylated dNTPs. The biotin-labelled genomic DNA fragments are extracted and sequenced [69].

For both animals and plants, 500–50,000 fresh cells are adequate, as opposed to MNase-seq or DNase-seq which requires at least 10^6^–10^7^ cells [16,62]. The high-resolution nature and small sample requirements of ATAC-seq make it an excellent tool for genome accessibility profiling; in fact, it was employed as a primary method for investigating the human epigenome in the ENCODE project [13,51].

For all above methods, at least two biological replicates are necessary to ensure the reproducibility. Based on the ENCODE Experiment Guidelines, the replicates must be independently derived from the same cell/tissue type/state. To be considered as reproducible data, the following criteria should be met: a) the number of mapped reads and the length of target lists from replicates should be within a factor of two of each other, and either b) 80% of the top 40% fraction of the target lists of the two replicates should overlap and same for the reciprocal, or c) there must be >75% of targets in common when all available reads of both replicates are compared (https://www.encodeproject.org/about/experiment-guidelines).

#### 1.2.2 Computational Approaches for Assessing Primary-order Chromatin

The key features of the pipeline developed to analyze high-throughput sequencing data for primary-order chromatin structure are outlined in Fig. 2. As shown in the figure, the analysis to discover chromatin accessible regions (right panel) follows steps common to the pipeline developed to identify higher-order chromatin structure. The following discussion focuses on features and tools specific for chromatin accessibility profiling. A summary of the computational tools employed for chromatin accessibility studies is shown in Table 2.

##### 1.2.2.1 Pre-processing and Alignment

The sequence reads are first quality checked and filtered to remove redundant reads and adaptors. At this stage, size selection is performed when required. In MNase-seq, smaller fragments (approximately 25–50 bp) represent transcription factor binding sites. ATAC-seq data containing specifically mapped fragments below 38 bp are removed, as 38 bp is the minimal distance between neighboring transposition sites generated by the Tn5 transposase [2]. In addition, reads originating from the mitochondrial genome are discarded. Subsequently, filtered reads are aligned to a user-defined reference genome using similar tools mentioned for higher-order structure assessment (Table 2). The minimal required number of sequencing reads for each method in the case of human is listed in Table 1; 150–200 million reads for MNase-seq, 20–50 million for DNase-seq and FAIRE-seq, and 100–160 million for ATAC-seq (Table 1).

##### 1.2.2.2 Preliminary Assessment

In the preliminary assessment of the sequencing results, composite plots are utilized to visualize read abundance as a function of the distance to a particular genetic feature. An increase in read abundance at positions corresponding to accessible regions indicates a good library. For example, transcription start sites (TSSs) have been demonstrated to be accessible chromatin locations. Hence, DNase-, FAIRE- and ATAC-seq data are expected to show an overall increase in abundance at these locations, whereas a decrease at TSSs is expected for MNase-seq data. For ATAC-seq specifically, an additional size distribution plot of inserts (i.e., fragments resulting from Tn5 transposition) can be generated using Picard tools (http://broadinstitute.github.io/picard/). The size distribution of inserts in a successfully prepared library depicts an array spanning five to six nucleosomal units. In addition to examining read abundance at the locations of certain genetic features, the visualization of read abundance across the entire genome provides a general read density profile. Publicly available genome browsers, such as Integrative Genomics Viewer (IGV) (see Table 2 for more tools), can be employed for this purpose. Among these browsers, IGV is one of the most powerful tools supporting the integrative analyses of genetic, epigenetic and expression data.

##### 1.2.2.3 Peak Calling

After preliminary assessment, mapped reads are used to detect open chromatin regions represented as “peaks” where the maximum number of reads are mapped. This “peak calling” step is perhaps the most critical step for chromatin accessibility profiling, revealing nucleosome-dense, closed regions (MNase-seq) or open chromatin regions (DNase-seq, FAIRE-seq, and ATAC-seq). In some cases, transcription factor binding sites can also be identified when small fragments (25–50 bp) are included in the sequencing library. For better clarity, specific analysis features and tools for each method are discussed individually below.

##### 1.2.2.4 Peak Calling for MNase-seq

For MNase-seq data, sequenced in single-end mode, the 5′ end of the mapped sequence for the forward or reverse strand represents the nucleosome border, and the midpoint or full nucleosome length can be identified by shifting the ends 73 bp [3] or extending the ends from 120 to 147 bp in the 3′ direction. For paired-end sequencing, the midpoint of the forward and reverse reads is assigned as the nucleosome midpoint. GeneTrack [4] employs Gaussian smoothing to generate a probability-based continuous map where nucleosome positions are assigned according to a user-defined exclusion distance between neighboring nucleosomes, whereas iNPS [20] detects nucleosomes from the first derivative of the Gaussian smoothed profile. DANPOS [19] enables the comparison of MNase-seq datasets and the identification of dynamic nucleosomes that respond to environmental conditions and development stages.

##### 1.2.2.5 Peak Calling for DNase-seq and FAIRE-seq

For DNase-seq data analyses, algorithms such as F-seq [15] and Hotspot [8,49] are specifically designed to manage the unique features of DNase-seq data. F-seq implements a smooth Gaussian kernel density estimation and has been implemented in combination with ChIP-seq in many studies to identify chromatin-accessible regions and tentative TF footprints. One unique feature of the Hotspot tool is that it reports statistical significance for identified DHSs. In addition, general peak-calling tools, such as MACS [106] and ZINBA [72], have been successfully employed as peak-calling software for DNase-seq data [101]. ZINBA uses a regression model to identify enriched regions, and regions within a defined distance are combined to form a broad region in which the positions of maximal sharp signals are identified using a shape-detection function. The model-based algorithm MACS was originally designed for ChIP-seq datasets but has been effectively applied to identify enrichment regions for DNase-seq data. MACS models the shift size and implements a Poisson distribution as a background model to detect enrichment. For FAIRE-seq data, the shift-size parameter should be set as the midpoint of the average size of sonicated fragments. ZINBA can also be used to detect enrichment for FAIRE-seq, as this technique shows better detection accuracy than MACS2 when the signal-to-noise ratio is low.

##### 1.2.2.6 Peak Calling for ATAC-seq

Paired-end sequencing is performed for ATAC-seq. Paired-end 50-cycle reads generally provide accurate alignments, and approximately 50 million mapped reads are sufficient for human samples [16]. The read start sites require adjustment because the Tn5 transposase binds as a dimer and inserts adaptors separated by 9 bp [2]. Generally, reads aligned to the + strand are offset by +4 bp, and reads aligning to the - strand are offset by −5 bp. ATAC-seq data can reveal both small transcription factor binding sites (indicated by narrow peaks) and larger regions of open chromatin (indicated by broad peaks). Broad peaks cover broad regions of enrichment, and localized/narrow peaks span small regions of approximately 50–500 bp. In most cases of chromatin accessibility profiling, the target open chromatin regions are a few kilobase pairs or longer and are presented as broad peaks. Open chromatin regions can be inferred from peaks using peak-calling tools. Common tools for the recognition of regions or peak calling for ATAC-seq include MACS [106], ZINBA [72], Hotspot [49], HOMER [41] and F-seq [14].

The MACS2 peak caller is a popular tool for ATAC-seq peak calling, as it can detect both narrow and broad peaks and considers the false discovery rate and noise. Similar to MACS2, ZINBA calls both broad and narrow regions of enrichment across a range of signal-to-noise ratios. Additionally, ZINBA accounts for factors that co-vary with the background or experimental signal. Hotspot can detect regions of enrichment of variable sizes and performs automatic normalization for large regions with elevated read levels, reflecting features such as high copy numbers. F-Seq is a Java package that continuously estimates the read density and identifies regions of higher density and was used to identify broad accessible regions in the ENCODE project. In contrast, HOMER was employed to call localized narrow peaks as HOMER was originally developed to identify short (8–12 bp) motifs for ChIP-seq analysis. An R module called “atac-seq” which implements the ATAC-seq pipeline of ENCODE, including F-seq, HOMER, and MACS2, with data visualization was recently made available (https://github.com/blikzen/atac-seq).

#### 1.2.3 Chromatin Accessibility Analysis

Accessible regions are determined based on peak-calling results. Positions of nucleosome occupancy can be assigned from MNase-seq data using various algorithms and TF-binding site tools, such as V-plots [18,103]. For DNase-seq, regulatory elements in open chromatin regions are identified using footprinting algorithms. Among these tools, DNase2TF [96] offers better detection accuracy and requires less computing time. In the analysis of ATAC-seq data, the positions of both nucleosome and TF-binding chromatin are identified using CENTIPEDE [68]. As algorithms exhibit various sensitivities and specificities, it is beneficial to analyze data using more than one tool because discrepancies in peak calling have been reported [53,95]. Additionally, cross-comparison of chromatin accessibility profiles generated using different methods to obtain consensus peaks or regions will be beneficial for downstream analyses.

### 1.3 Profiling the Chromatin Hierarchy in Single Cells

The use of 3C-based methods in large populations of cells generates population-averaged maps of chromosomal contact frequencies. To understand the cell-to-cell variability in the chromosome architecture, Flyamer and co-workers developed an *in situ* Hi-C approach [34]. Conventional Hi-C methods include biotin labeling and enrichment for ligated fragments, which limits fragment retrieval; hence these steps were omitted in the *in situ* Hi-C protocol. These authors reported up to 1.9 × 10^6^ contacts per oocyte in mice after filtering, yielding 1–2 orders of magnitude more contacts than previously reported single-cell Hi-C data [66]. The same study showed that loops and compartments were formed by distinct mechanisms. In another study, Ramani and colleagues developed a single-cell combinatorial Hi-C (sciHi-C) index method, in which combinatorial cellular indexing was applied to capture the chromosome conformation [70]. In combination with other single-cell studies of methylomes and transcriptomes, comprehensive details of the interplay between these hierarchical levels can be obtained.

A non-3C-based method, called genome architecture mapping (GAM), combine ultrathin cryosectioning with laser microdissection and DNA sequencing to capture three-dimensional proximities between genomic loci without the ligation step [11]. Based on the assumption that physically proximal loci are found more frequently in the same thin nuclear section than distant loci, GAM infers the chromatin spatial structure by determining the presence or absence of genomic loci in a set of single slices (one slice per nucleus) from a population of nuclei. The co-segregation of loci among a large collection of nuclear profiles is used to create a matrix that is further analysed to identify chromatin contacts genome-wide. Notably, in mouse embryonic stem cells the identified contacts were enriched for regions that are highly transcribed or contain super-enhancers.

Similarly, the profiling of chromatin accessibility often requires a large population of cells. The chromatin landscapes of each cell type are lost when only the average profile is assessed. Hence, the need for epigenetic investigation within complex and heterogeneous tissues drives the development of accessibility profiling techniques at single-cell resolution [6,22,82].

An investigation of open chromatin regions in single cells has been demonstrated based on a modified DNase I protocol [48]. Using the described single-cell DNase-seq (scDNase-seq) technique, a resolution of 300,000 mapped reads per single cell was achieved. Comparative analysis among individual cells revealed that constitutive DHSs reside in highly expressed gene promoters and enhancers associated with multiple active histone modifications. In addition to DNase-seq, two single-cell level ATAC-seq methods have also been demonstrated recently. The first method employs a “combinatorial indexing” strategy in which tagmentation is performed on 96 reactions involving a few thousand nuclei, introducing a unique barcode to each reaction. The 96 reactions are subsequently pooled and split, prior to a second round of tagging via PCR. This two-step process results in a unique barcode combination for each individual cell [24]. In the second method, a microfluidics device is used to encapsulate individual cells within aqueous droplets, in which the transposition reaction occurs [17]. This approach results in a large increase in resolution compared with the combinatorial indexing method, with an average of 70,000 reads per cell. scATAC-seq shows great potential for elucidating the cellular variation of the chromatin landscape [17,24], and the assessment of chromatin accessibility requires a different computational analysis method. One such method was described by Buenrostro et al. [17]; a set of chromatin peaks was first identified from the aggregate accessibility track. The fragment abundance at these peaks was adjusted based on the expected abundance within individual cells. The cellular variance was subsequently calculated as a “variability” score, which was corrected against the background signal resulting from technical and sampling errors. Further development in single-cell chromatin profiling techniques will advance our understanding in the role of chromatin accessibility in physiological heterogeneity related to vital biological processes.

### 1.4 Biological Relevance of Chromatin Organization

The higher-order organization of chromatin has implications in major cellular functions [10]. For example, in mice and humans the efficiency of DNA repair depends on the higher-order chromatin structure [37,65]. Furthermore, chromosomal abnormalities in the form of translocations and aneuploidy are a general hallmark of cancer cells [64]. The genes associated with chromosomal translocations in human lymphomas are in the physical proximity of each other and are located towards the nuclear interior. The translocations depend on the “higher-order spatial organization” of the genome, rather than the sequences of the genes involved [76]. Similarly, the primary-order chromatin structure regulates several biological functions. Understanding the dynamics of the chromatin landscape provides clues about disease development and cell differentiation. For example, changes in accessibility of specific transcription factors were identified as a determining factor for cancer development [25]. In a chromatin accessibility profiling study using scDNase-seq, thousands of tumor-specific DNase I-hypersensitive sites were identified [48]. It was found that these hypersensitive sites are highly associated with cancer development.

### 1.5 Integrative Approaches for Assessing the Chromatin Hierarchy

Other high-throughput technologies have been combined with chromatin conformation capture methods for validating chromatin interactions and examining their biological relevance. Chromatin interaction analysis based on paired-end tag (ChIA-PET) sequencing combines ChIP with chromatin conformation capturing techniques, potentially facilitating the identification of chromatin contacts with sites bound by a protein of interest [26]. Imaging tools such as fluorescent *in situ* hybridization (FISH) and electron microscopy (EM) can be combined with 3C-based technologies to overcome their limitations in terms of resolution and scale [85]. Imaging can also be combined with chromatin accessibility profiling. Chen et al. [21] developed “ATAC-see” that employs bifunctional Tn5 transposome with fluorescent adaptors to mark the accessible genome *in situ*. Moreover, a recently developed method called NicE-seq (nicking enzyme assisted sequencing) has been demonstrated for its capacity to identify open chromatin regions, and this technique's potential to visualize open chromatin when coupled with fluorescent-labeled dNTPs has been proposed [69].

Integrating chromatin accessibility data obtained from ATAC-seq and other techniques with RNA-seq and ChIP-seq enables researchers to elucidate associations of chromatin states with gene expression and regulation. For example, by correlating the accessibility map generated using ATAC-seq with RNA-seq data, candidate *cis-*regulatory elements responsible for cell differentiation were identified [1]. Likewise, the combination of the newly developed scATAC-seq method and ChIP-seq analysis revealed *trans-*regulatory elements that induce or suppress cell-to-cell heterogeneity [17]. In another single-cell profiling study using scATAC-seq, the integration of RNA-seq data led to the identification of a cell surface marker that co-varies with chromatin accessibility changes associated with cancer cell heterogeneity [61]. These integrative approaches demonstrated that the combination of different techniques provides complementary information that help elucidate the regulatory mechanisms of various cellular functions.

## 2 Summary and Outlook

Cellular functions are often a consequence of the coordinated action of the chromatin hierarchy. Integrating chromatin studies of higher-order and primary-order structures sheds light on the potential link between these two levels of chromatin structures. For example, it has been demonstrated that genomes from bacteria to mammals segregate into domains wherein segments of DNA preferentially interact, and this preference is associated with epigenetic signatures of the primary chromatin structure. This link between primary-order chromatin accessibility and higher-order chromatin compartmentation was also reported in a single-cell level chromatin profiling of mammalian cells using scATAC-seq [17]. Clearly, the interactions of chromatin in a three-dimensional space depend on its primary structure and associated epigenetic marks. However, the underlying mechanism remains unclear.

Several limitations challenge the assessment of the higher-order chromatin hierarchy. First, experimental steps including crosslinking, chromatin fragmentation, biotin labeling and ligation introduce biases that complicate the interpretation of detected interactions. Moreover, long-range chromatin interactions and the principles of chromatin dynamics require more accurate, sensitive and reproducible methods. Some of these issues can be addressed by integrating data from multiple biological replicates because higher reproducibility indicates higher reliability and/or stability of the detected interactions. Binning is a critical step that increases the signal of the interaction frequency. Furthermore, most multicellular organism are diploid but the current pipelines for chromatin conformation prediction consider the genome as haploid. To address the issue, single-nucleotide polymorphisms (SNPs) and insertion deletion polymorphisms (Indels) can serve as markers separating sister chromatids; these markers can be incorporated into the computational pipeline to increase the accuracy of chromatin conformation.

For primary order structure analyses, future opportunities and challenges include the incorporation of chromatin interactions and accessibility profiles with genetic and epigenetic features to elucidate the intricate regulatory network. Such analyses should also include the association of chromatin interactions and accessibility with allele specificity for functionally relevant SNPs. Combining the results from genome-wide accessibility profiles with quantitative trait locus studies can aid the identification of disease phenotypes. These challenges highlight the need for robust computational tools tailored specifically towards an integrative approach. Furthermore, as single-cell analysis contributes to the clarification of relationships between gene expression and epigenetics, we foresee that this technique will provide new insights into the role of chromatin accessibility in physiological heterogeneity related to cell differentiation, development, health and disease. However, low sequencing coverage is a major issue for single-cell-level techniques which therefore require advancement in sequencing techniques.

## Abbreviations

TAD: Topology associating domains
3C: Chromosome conformation capture
4C: Circular chromosome conformation capture
5C: Chromosome conformation capture carbon copy
LMA: ligation-mediated amplification
DI: Directionality index
ID: Insulation index
MNase-seq: micrococcal nuclease sequencing
FAIRE: formaldehyde-assisted isolation of regulatory elements
ATAC-seq: assay of transposase-accessible chromatin sequencing
DHS: DNase I hypersensitive site
ChIA-PET: chromatin immunoprecipitation with paired-end tag sequencing
FISH: fluorescence *in situ* hybridization
EM: Electron microscopy
SNP: single-nucleotide polymorphism
Indel: insertion deletion polymorphism

## Author contributions

All authors contributed to the conception and writing of this manuscript.

## Conflict of Interest

The authors declare no conflicts of interest.
